# A multi-faceted approach to the study of the relationship between self-esteem and the self-prioritization effect

**DOI:** 10.1007/s00426-026-02365-8

**Published:** 2026-08-25

**Authors:** Lorenzo Atzeni, Luigi Castelli, Mario Dalmaso, Giovanni Galfano, Barbara Penolazzi

**Affiliations:** 1 Department of Developmental Psychology and Socialization, University of Padova, Via Venezia, 8, Padova, I- 35131 Italy; 2 Department of Life Sciences, University of Trieste, Trieste, Italy

## Abstract

A key aspect of self-referential processing is that self-related stimuli tend to receive preferential processing in several cognitive domains. The so-called matching task has been introduced to quantify this bias by means of arbitrary self–other associations, aimed to minimize potential confounds related to stimulus familiarity. In this paradigm, participants learn associations between neutral shapes and social labels (e.g., self vs. stranger) and then perform a perceptual matching task. The self-prioritization effect (SPE) refers to the observation that participants are faster and more accurate in self-matching trials compared to matching trials concerning the “other”. Whether individual differences in implicit and explicit self-esteem can modulate the magnitude of the SPE is still a matter of debate. The present study aimed to address this question. Using a longitudinal design to assess the temporal stability of the effects, participants completed the Self-Esteem Implicit Association Test and the Name Letter Test as measures of implicit self-esteem, and the Rosenberg Self-Esteem Scale (RSES) as a measure of explicit self-esteem, alongside the matching task. The results confirmed the reliability of the SPE over time. Furthermore, unlike measures of implicit self-esteem, explicit self-esteem showed significant positive correlations with the SPE, with RSES scores being able to predict the magnitude of the SPE. Importantly, these associations were observed both within the same testing session and between sessions. These findings suggest that explicit self-evaluation is associated with the magnitude of self-prioritization. The possible implications of this pattern for theoretical accounts of self-referential processing are discussed.

## Public significance statement

Prioritizing information about oneself is a pervasive feature of human cognition, shaping attention, memory, and social behavior. In a three-session study, we found that higher explicit self-esteem was positively correlated with the tendency to perceptually prioritize self-related information over stimuli related to the “other”. This suggests that the way we consciously perceive and evaluate ourselves can shape even rapid forms of self-related processing, with potential implications for understanding fluctuations in self-referential processing.

Cognitive psychologists have repeatedly observed that self-related information is prioritized over the vast majority of stimuli that permeate our complex environments. One of the earliest findings related to this robust cognitive bias was provided by Moray (1959). In particular, he observed that during dichotic listening, even when participants attempted to focus elsewhere, it was almost impossible for them to inhibit the automatic shift of attention triggered by hearing their own name. Later, a similar effect was found with a variety of different stimuli, such as one’s own face (e.g., Sui et al., 2006; Tong & Nakayama, 1999), one’s own voice (Candini et al., 2014), and one’s own body parts (e.g., Devue et al., 2007; Frassinetti et al., 2009), which are prioritized and tend to capture attention more effectively with respect to other stimuli. However, this tendency may simply reflect a bias towards highly familiar information. To address this possible confound and rule out the role of familiarity, Sui et al. (2012) have developed an ad-hoc experimental paradigm based on stimuli with a neutral valence, reasonably not contaminated by unbalanced familiarity. Specifically, in the matching task proposed by Sui et al. (2012), participants learn novel episodic associations between neutral stimuli (i.e., geometrical shapes) and social labels (e.g., either the self or a stranger). Later, participants are instructed to decide whether the presented shape-label pair matches the previously learned associations. The self-prioritization effect (SPE) refers to the finding that self-related matching trials yield faster response times (RTs) and greater accuracy than other-related matching trials.

So far, the SPE has been extensively studied and has inspired numerous research studies (see, e.g., Dalmaso et al., 2024, 2026; Golubickis & Macrae, 2023). These have pursued two primary objectives: (a) exploring potential interpretations for the occurrence of the SPE and (b) examining factors that influence this phenomenon. Regarding the first issue, Sui et al. (2012) proposed that neutral stimuli acquire social salience upon association with the self, which, in turn, enhances their processing at early perceptual stages (this interpretation also converges with evolutionary theories that account for “self-effects” as reflecting an adaptive mechanism that could promote self-survival; Sui et al., 2012, but see also Pauly & Wentura, 2025). Neuroimaging studies have identified a neural circuit (including the ventromedial prefrontal cortex and the left posterior superior temporal sulcus) involved in the processing of self-relevant stimuli during the matching task (Sui et al., 2013), and behavioural evidence indicates that self-associated shapes can capture and hold attention more effectively than shapes associated with the other (Dalmaso et al., 2019).

More recently, Pauly and Wentura (2025) have reinterpreted the SPE through the lens of the polarity correspondence principle (Proctor & Cho, 2006). From this perspective, the SPE would emerge due to the correspondence of positive polarity shared by the “self” label, the associated shape, and the affirmative match response (“yes”) that occurs in self-matching trials. By contrast, stimuli assigned to the “other” category would possess a negative polarity, which conflicts with the positive coding of the “yes” response. Pauly and Wentura (2025) conducted several experiments and showed that, when instructions were changed so that participants had to respond “no” to positive matches, the SPE vanished. Notably, however, the SPE survived this manipulation in one study, suggesting that the polarity correspondence principle may account for part of the effect but cannot fully explain the phenomenon.

As concerns factors that influence the SPE, several studies have explored variables that may affect its emergence. Among these, one of the most relevant to the aim of the present research is the role of individual mood and emotional valence of self-related stimuli in the emergence of the SPE. In this regard, Sui et al. (2016) performed a study in which they elicited a negative mood through the use of either melancholic music or exposure to self-referential negative statements, after which participants performed the matching task. The results showed that the SPE was reduced when participants underwent a negative mood induction, suggesting that the SPE is modulated by one’s own emotional state. Among the different interpretations, the authors suggested that self-related stimuli tend to evoke a positive emotional response that boosts perceptual matches, and that a negative mood weakens this response, thereby diminishing the self-advantage. Converging evidence comes from studies that directly manipulated the valence of the stimuli associated with the self. Hu et al. (2020) manipulated the valence of the self and the others using a variant of the matching task. The participants learned an association between a shape and identity-based valence categories, notably either the “good” or “bad” part of the self or other (e.g., circle/good-self, triangle/good-other, diamond/bad-self, square/bad-other). The results revealed a consistent prioritization of stimuli associated with the “good-self”, suggesting that positive aspects of self-concept play a critical role in enhancing perceptual efficiency. Crucially, this advantage emerged specifically for the good-self relative to the good-other, whereas no comparable difference was found between the bad-self and the bad-other, ruling out a general aspecific valence account. On the same line, Constable et al. (2021) demonstrated that SPE was significantly enhanced when participants associated happy faces with the self, compared to when they associated sad faces, suggesting that the emotional valence of self-associated stimuli plays a critical role in self-integration processes. In a related vein, Vicovaro et al. (2022) had participants performing a matching task in which symmetrical and asymmetrical geometric shapes were randomly associated to the self and the other. The authors found that the SPE emerged only in the condition in which the self was associated with the symmetrical shape. The results were interpreted by assuming that symmetrical stimuli have a positive valence (Makin et al., 2012) and are better associated to the self than asymmetric shapes.

Building on this evidence, some authors have shifted the focus from the valence of the stimuli paired with the self to the valence that individuals implicitly or explicitly assign to themselves, exploring whether self-esteem (SE) contributes to the emergence of the SPE. This view is supported by the implicit positive-association theory of self-advantage (Ma & Han, 2010), according to which self-related stimuli may acquire a processing advantage through their association with positive valence. If the SPE is likewise supported by the positive valence habitually associated with the self, individual differences in self-esteem should be expected to modulate its magnitude.

Early evidence by Schäfer and Frings (2019) indicated no significant correlation between explicit SE, measured with the Rosenberg Self-Esteem Scale (RSES; Rosenberg, 1989), and the SPE, suggesting that the two constructs may be unrelated. Recently, this null result has been replicated by Mou and Ding (2026) using a different stimulus administration, namely a voice-label matching task in which unfamiliar voices were arbitrarily paired with the self or others. Hence, explicit SE did not correlate with the SPE inferred from neutral vocalizations. However, when the authors further manipulated the prosodic valence of the voices, explicit SE positively correlated with the tendency to prioritize happy self-associated voices over neutral voices. In particular, higher explicit SE was selectively associated with stronger prioritization of positively valenced self-voices, while leaving unaffected the prioritization of neutral self-voices. Crucially, a different picture emerged when the focus shifted to children. In this regard, Tan and Li (2025) examined whether self-evaluative processes predicted the strength of the SPE in 9- to 11-year-olds, simultaneously measuring both explicit and implicit SE, measured with the RSES and a variant of the SE Implicit Association Test (SE-IAT; Greenwald & Farnham, 2000), respectively. Their findings revealed that explicit SE positively predicted the magnitude of the SPE, whereas implicit SE did not. Further insight into the relationship between implicit SE and the SPE comes from Hobbs et al. (2023), who explored self-processing in adults experiencing varying levels of depression. By combining the matching task with an SE go/no-go task, the authors found that depression severity was associated with altered implicit self-evaluations but was not associated with the magnitude of the SPE. Although the study was not designed to directly test the SE–SPE association, these findings provided preliminary, albeit indirect, support for the relative independence between the SPE and individual differences in implicit self-evaluation. Taken together, the available evidence presents a complex picture in need of further investigation.

The present study aimed to deepen our understanding of the relationship between the SPE and both explicit and implicit SE, examining whether and how individual differences in self-worth influence the emergence and the strength of the SPE. To this end, we introduced two important variations with respect to the previous studies (Mou & Ding, 2026; Schäfer & Frings, 2019; Tan & Li, 2025). First, we implemented a more comprehensive assessment of SE, including well established measures of implicit SE, namely, the SE Implicit Association Test (SE-IAT; Greenwald & Farnham, 2000) and the Name Letter Test (NLT; Nuttin, 1985), which have not been previously used with adults in the context of the SPE. According to the dual-process theory, explicit and implicit SE should reflect two relatively distinct components of the self-evaluative process (Hofmann et al., 2005; Strack & Deutsch, 2004). The former refers to high-order, propositional self-evaluations accessible to conscious processes, while the latter refers to more automatic self-evaluations. Examining both components simultaneously allows for a more comprehensive assessment of how self-evaluation, at different levels of processing, is potentially related to the SPE. The second novel contribution with respect to previous works relied on the adoption of a longitudinal design with three measurement sessions which, in turn, allowed us to examine the temporal stability of both the SPE and its potential correlations with SE measures. This design enabled us to assess the test-retest reliability of the SPE, addressing recent concerns about the psychometric properties of self-matching task measures (see Liu et al., 2025).

Regarding the theoretical implications of the present study, the pattern of results may lead to distinct theoretical perspectives depending on the specific associations observed between SE and the SPE. Specifically, different scenarios may be found. First, the absence of any association with both explicit and implicit SE would be consistent with the theoretical account that frames the SPE as a low-level, perceptual process, independent of how individuals evaluate themselves. However, the correlational design of the present study would not allow us to draw strong conclusions in that scenario, since an absence of correlation may also suggest that the SPE is relatively independent of these particular self-evaluative constructs, rather than constituting direct evidence for a purely perceptual account of the effect.

Second, a significant association with SE measures would indicate that higher-order self-evaluations can, at least partially, modulate the SPE in a top-down fashion, in line with the findings of Tan and Li (2025). The specific approach implemented in the present study enables us to address the eventual relative contribution of both implicit and explicit SE measures. The second scenario, entailing some form of association between SE and SPE, would suggest that self-related stimuli are perceived as more salient depending on the subjective self-view, indicating that the relevance of self-related stimuli is dynamically influenced by the individual’s subjective self-evaluation.

## Methods

### Participants

Data collection took place across three sessions, with intervals of approximately one week between sessions.

No formal a priori power analysis was conducted prior to data collection. The target sample size was instead guided by previous research addressing the relationship between explicit SE and the SPE, and we aimed to recruit a sample approximately as large as those included in that literature (e.g., Schäfer & Frings, 2019; *N* = 103). A total of 200 undergraduate psychology students from the local university were invited to take part in the study. A total of 149 undergraduate psychology students from the University of Padova (Italy) volunteered to participate in the first session; 119 took part in the second session, and 97 in the third session. All participants read and accepted the informed consent approved by the Ethics Committee for Psychological Research at the local university (Protocol 987-a; dated January 24, 2025). The study was conducted entirely online: experimental tasks were programmed in PsychoPy (version 2022.2.4; Peirce et al., 2019) and administered via Pavlovia. Participants were excluded from analyses if they had duplicate or unresolvable identification codes across sessions, or due to methodological criteria detailed below. After applying these criteria, the final sample was *N* = 146 in the first session. In the second session, sample size depended on the measure: *N* = 112–113 for the SE measures (SE-IAT/RSES, respectively) and *N* = 106 for the matching task (SPE), following task-specific exclusions. In the third session, the final sample for the matching task was *N* = 84. However, not all participants who took part in a given session had necessarily participated in previous ones, and not all participants from earlier sessions returned for subsequent ones. Specifically, analyses involving first- and second-session measures were conducted on 84 to 90 participants, depending on the specific measures considered, while analyses involving second- and third-session SPE scores were based on 65 participants (see Table 1). Task-specific exclusion criteria and exact sample sizes for each analysis are reported in the Results section. Since the number of available participants decreased across sessions due to the longitudinal design and task-specific exclusions, we also conducted a sensitivity power analysis using the pwr package in R (Champely, 2020). This analysis estimated the smallest correlation that could be detected with 80% power at *α* = 0.05 (two-tailed), given the realized sample sizes of the main correlational analyses. Across the analyses directly examining the relationship between self-esteem and the SPE, sample sizes ranged from *N* = 106 to *N* = 66, corresponding to minimum detectable correlations ranging from *r* =.27 to *r* =.34. All anonymized raw data and scripts can be found at https://osf.io/tmk5b/overview?view_only=59482ae7d4d44e5dad3765e5b91f7894.

**Table 1:** Participant numbers and exclusions across sessions

Session	Measures administered	*N* collected	Exclusions	*N* final
S1	SE-IAT, NLT, RSES	149	3 (duplicate/unresolvable ID codes)	146
S2 – SE-IAT	SE-IAT	119	6 (duplicate ID codes); 1 additional (see SE-IAT scoring)	112
S2 – Matching task	Matching task	119	6 (duplicate ID codes); 7 additional (< 50 valid trials)	106
S2 – RSES	RSES	119	6 (duplicate ID codes)	113
S3 – Matching task	Matching task	97	8 (duplicate ID codes), 5 additional (< 50 valid trials)	84

*SE-IAT* Self-Esteem Implicit Association Test, *NLT* Name Letter Test, *RSES* Rosenberg Self-Esteem Scale. S1, S2, and S3 refer to the first, second, and third testing sessions, respectively. Longitudinal analyses were conducted on partially overlapping subsamples: S1–S2 analyses ranged from *N* = 84 to 90 depending on the specific measures considered; prospective analyses predicting the SPE at S3 ranged from *N* = 66 to 69; S2–S3 analyses of SPE stability were based on *N* = 65. Not all participants who took part in a given session had necessarily participated in previous ones, and not all participants from earlier sessions returned for subsequent ones

### Procedure

The study employed a longitudinal design with three measurement sessions. Participants completed the tasks using their own personal computers in a self-selected quiet environment. During the first session, participants completed measures of implicit (i.e., the SE-IAT, the NLT) and explicit (i.e., the RSES) self-esteem. During the second session, participants completed the matching task assessing the SPE, followed by the SE-IAT and the RSES. During the third session, participants completed only the matching task. The arrangement of tasks across sessions was guided by specific methodological considerations. In the first session, SE measures were administered prior to any exposure to the matching paradigm, with the goal of gathering baseline indices of implicit and explicit SE collected independently from any self-referential task processing. This ensured that cross-session predictive relationships between SE and the SPE could be examined using scores not contaminated by the cognitive demands of the matching task. In the second session, the SE-IAT and RSES were administered again, this time immediately after the matching task. This served two purposes: first, it enabled test-retest reliability of SE assessment across sessions; second, it provided SE scores collected within a measurement context in which self-referential processing had been actively engaged, allowing us to examine whether concurrent SE–SPE associations differed from those observed with baseline SE scores at the first session. Including the matching task during both the second and third session allowed us both to assess the temporal stability of the SPE and to test whether SE scores measured at the second session prospectively predicted SPE magnitude at the third session. As regards the implicit SE measures, because repeating both the SE-IAT and the NLT would have considerably lengthened the second session, we decided to administer only the SE-IAT, for both practical and methodological reasons. Indeed, the SE-IAT is the implicit measure most directly comparable with previous studies addressing the SE–SPE relationship (e.g., Tan & Li, 2025), and its scoring procedure is less likely to be affected by participants recalling their previous responses as compared to the NLT. Moreover, the NLT requires rating the entire alphabet on a limited scale. The NLT was nonetheless included at the first session as an additional, methodologically independent index of implicit SE within the baseline measurement context. Lastly, SE was not reassessed at the third session because a further assessment was not among the primary objectives of the present study. The third session, as mentioned before, was included to evaluate the temporal stability of the SPE and to test whether SE measured at the previous sessions prospectively predicted the SPE, and both aims could be addressed with the SE scores already collected at the first and second sessions.

**Fig. 1:**
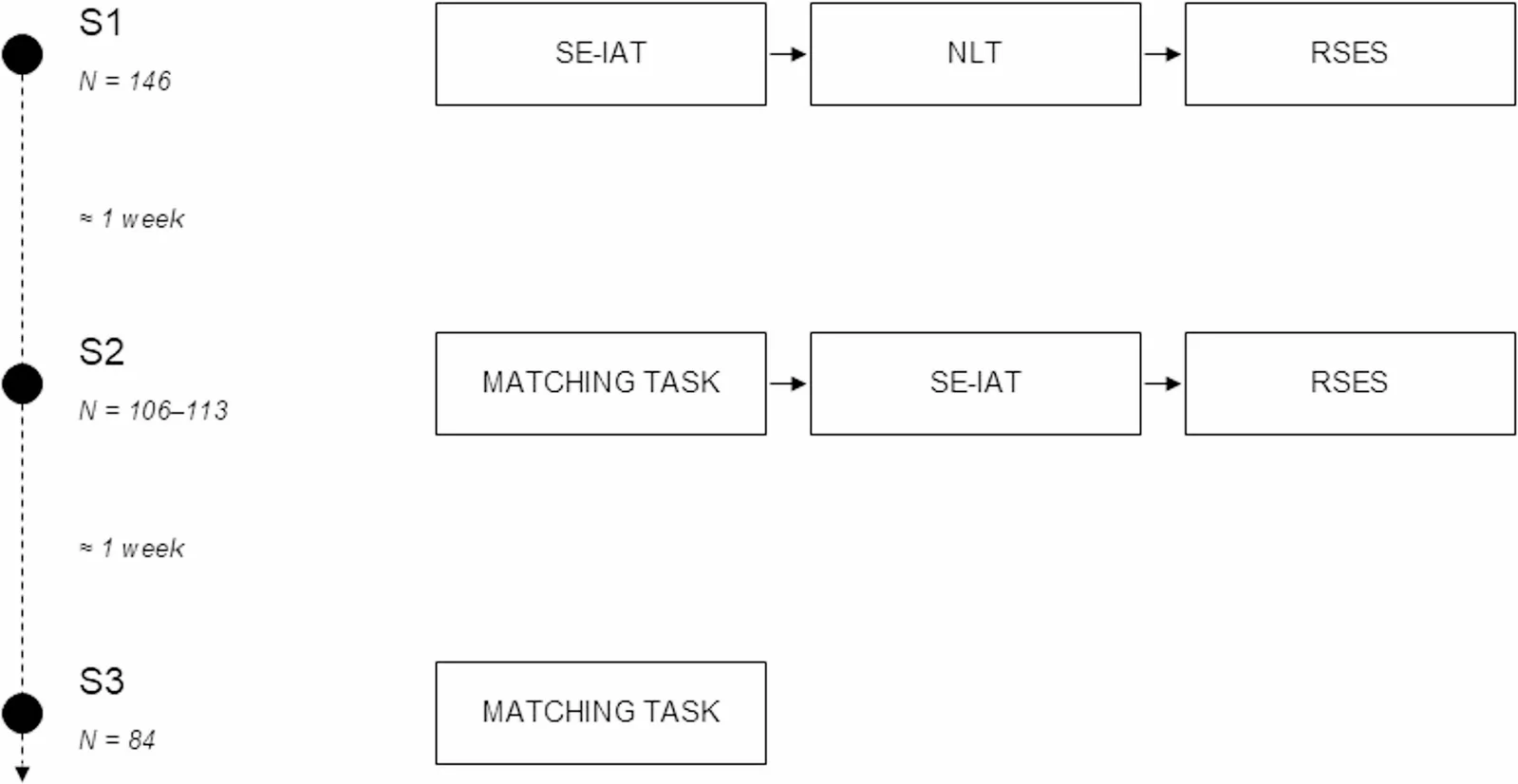
Overview of the longitudinal study design. S1, S2, and S3 refer to the first, second, and third testing sessions, respectively. During the first session, participants completed the Self-Esteem Implicit Association Test (SE-IAT), the Name Letter Test (NLT), and the Rosenberg Self-Esteem Scale (RSES). During the second session, participants performed the matching task, followed by the SE-IAT and the RSES. During the third session, participants completed the matching task only

As anticipated earlier, experimental sessions were scheduled approximately one week apart (see Fig. 1). The order of tasks within each session was fixed. Within each session, implicit SE measures were administered before the explicit measure. This order was chosen because implicit measures may be more easily influenced by a preceding explicit self-evaluation than the reverse. Specifically, reflecting on one’s self-worth through the RSES could carry over to subsequent implicit assessments, whereas the opposite pattern seems less likely. Counterbalancing task order would have undermined this rationale, placing the explicit measure before the implicit ones for some participants. On the same line, the order of the matching task and the SE measures within the second session was fixed rather than counterbalanced. The matching task was administered at the beginning of the session to prevent the cognitive demands of explicit and implicit self-evaluation from contaminating the SPE. At both the second and third sessions, participants completed the matching task using the same shape–label assignments. The assignments were kept identical across sessions to avoid interference from the previously learned associations, which would have introduced additional noise in the cross-session comparison. The learning phase was nonetheless repeated in full at the third session, since the associations could not be assumed to remain active after a one-week interval. Accordingly, the test–retest analysis reported in the present study reflects the stability of the SPE, that is, the extent to which the magnitude of self-prioritization is reproducible across sessions, rather than the spontaneous persistence in memory of the learned associations. At the beginning of each session, participants were provided with standardized instructions emphasizing the importance of responding as quickly and accurately as possible. Each session lasted approximately 20–30 min.

### Materials and tasks

#### Self-Esteem implicit association test

Implicit SE was assessed using a SE-IAT (Greenwald & Farnham, 2000). Before starting the task, participants were instructed to categorize target words into specific categories by pressing either the “a” or “l” keys of the keyboard.

**Fig. 2:**
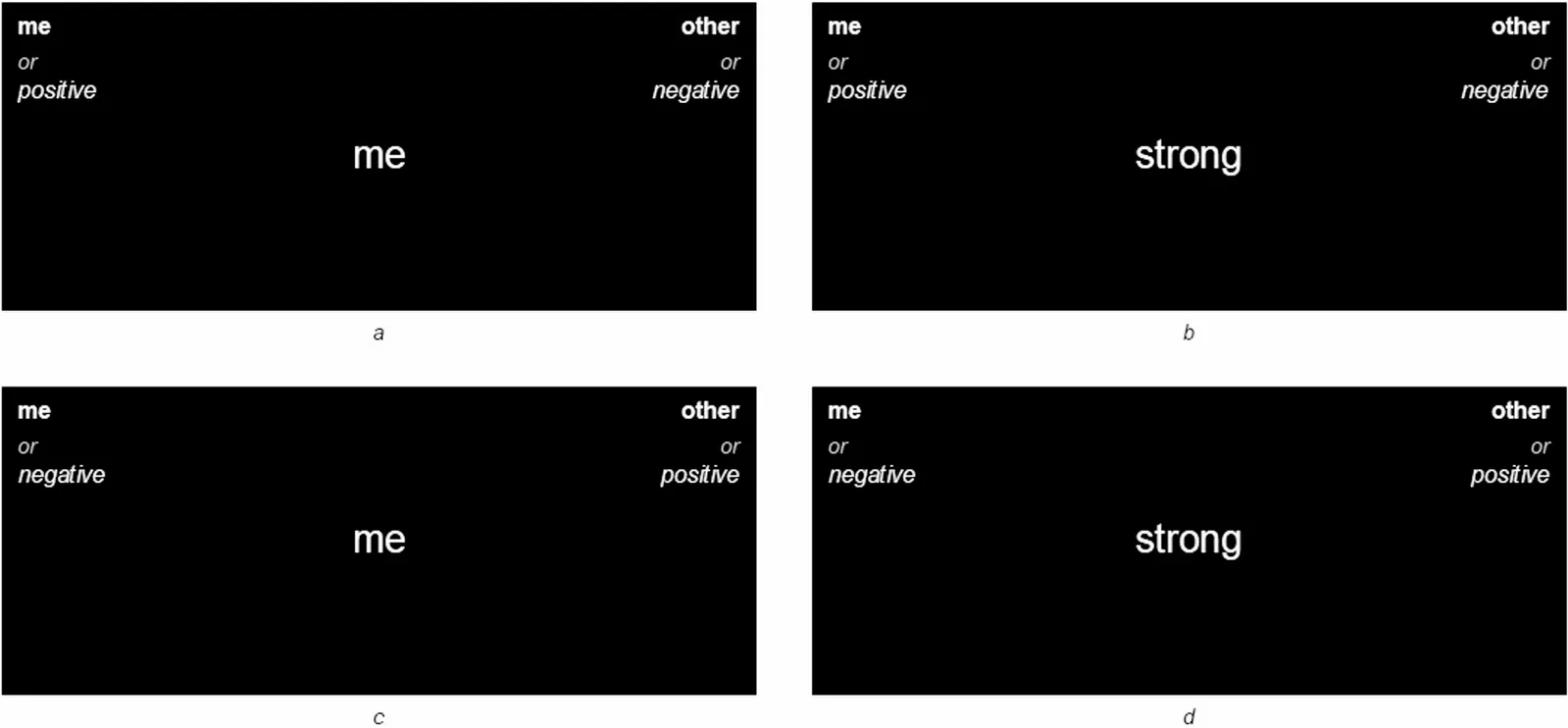
Schematic illustration of trials from the Self-Esteem Implicit Association Test. Panels (**a**) and (**b**) depict example trials from the congruent critical block, in which self-related pronouns and positive attributes were mapped onto the same key. Panels (**c**) and (**d**) depict example trials from the incongruent critical block, in which self-related pronouns and negative attributes shared the same key. Category labels appeared at the top-left and top-right of the screen throughout each block; the target stimulus was presented at the centre. Stimuli are shown here in English translation; the task was administered in Italian

In the first block, 10 pronouns (5 self-related and 5 other-related) appeared in randomized order at the centre of the screen (font: Open Sans, letter height: 0.05 height units). Participants categorized these pronouns into the corresponding categories—“me” and “other”—with no response time limit. In the second block, participants categorized 10 randomly presented attributes (5 positive and 5 negative; displayed in Open Sans font with a letter height of 0.05 height units) into two distinct categories—“positive” and “negative”—using the assigned keys. In the third block (congruent critical block), participants categorized both pronouns and attributes using the same key, specifically pairing self-related pronouns with positive attributes and other-related pronouns with negative attributes. In the fourth block, the key assignments were reversed, and participants practiced this new association by categorizing only attributes. In the fifth block (incongruent critical block), participants categorized pronouns and attributes with the reversed association: self-related pronouns were paired with negative attributes, and other-related pronouns with positive attributes (see Fig. 2).

In each block, the labels referring to the target categories were displayed continuously at the top left and top right of the screen (− 0.6 and 0.6 height units horizontally, 0.4 height units vertically), in Open Sans font with a letter height of 0.05 height units. Trials in all blocks had no response time limit, with each trial followed by a 1000-ms intertrial interval (ITI). The first, second, and fourth blocks consisted of 20 trials each, while the third and fifth blocks consisted of 40 trials each. Both critical blocks were preceded by 20 practice trials. For each incorrect response, a feedback signal (a red cross) appeared for 1000 ms.

#### Name letter test

Implicit SE was additionally measured using the NLT (Nuttin, 1985). Each letter of the alphabet was presented individually at the centre of the screen in random order. Participants rated each letter based on their immediate feelings of liking, using a 9-point Likert scale ranging from 1 (dislike extremely) to 9 (like extremely). Implicit SE is calculated by comparing the value attributed to the initial letters of one’s first name and surname with that of all other letters. Each trial was separated by a 300-ms ITI. At the end of the task, participants were asked to enter the initials of their first name and surname.

#### Rosenberg self-esteem scale

Explicit SE was measured using the Italian version of the RSES (Rosenberg, 1989; Italian adaptation by Prezza et al., 1997). The RSES comprises 10 items assessing global self-worth. Total scores range from 0 to 30, with higher scores indicating higher explicit SE. The scale was administered without time limits. Participants were informed that statements would appear at the centre of the screen, and they were asked to rate their agreement with each statement on a 4-point scale ranging from “Strongly disagree” to “Strongly agree.”

### Matching task

**Fig. 3:**
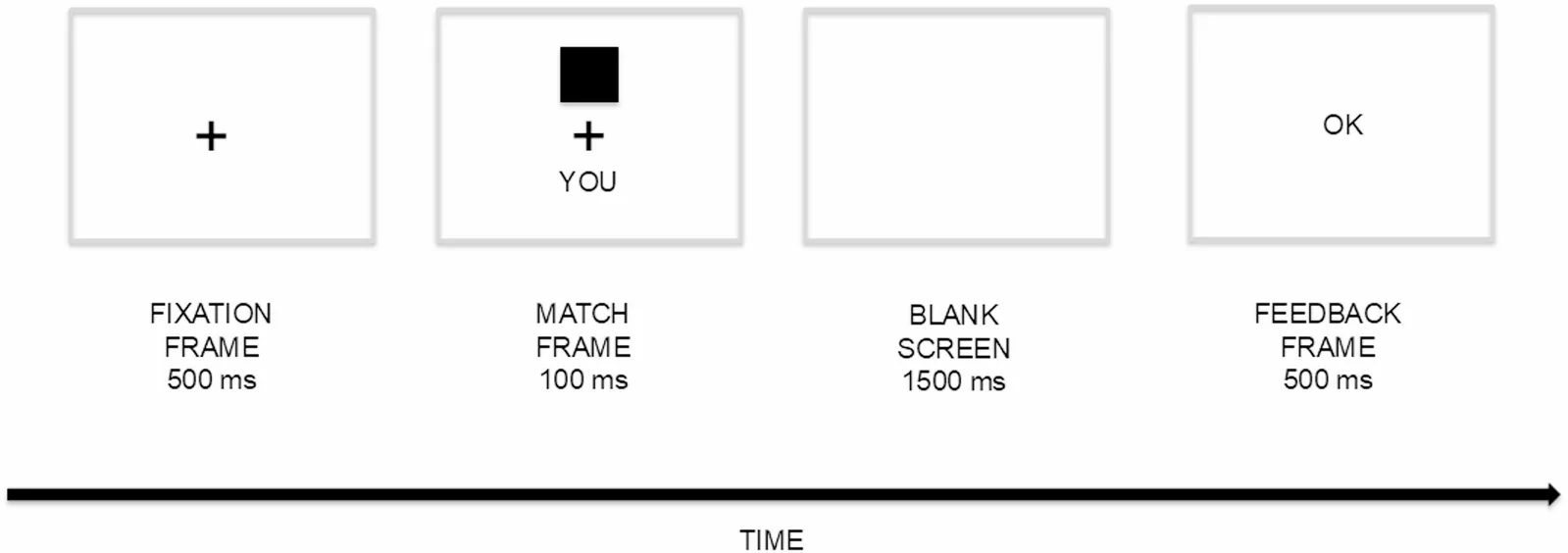
Schematic illustration of a trial in the matching task. Each trial began with a fixation cross (500 ms), followed by the simultaneous presentation of a geometric shape and an identity label (100 ms). A blank screen (1500 ms) was then displayed, during which participants were required to indicate whether the shape-label pair matched the previously learned associations by pressing the corresponding key. Feedback (“OK” for correct responses) was presented for 500 ms. Labels were presented in Italian language

The SPE was assessed using the matching task adapted from Sui et al. (2012). During the learning phase, participants were instructed to read and memorize a shape–label association displayed at the centre of the screen for 40 seconds: “YOU are the square; the triangle is the STRANGER” (font: Arial, 0.08 height units). Assignment to the two shape–label mapping conditions was counterbalanced across participants: half associated the self with the square and the stranger with the triangle, while the other half learned the opposite association. In the matching phase, participants were instructed to press either the “a” or the “l” key of the keyboard to indicate whether or not the displayed shape–label matched the previously learned association. The response-key assignment was fixed across participants and did not vary as a function of the specific shape–label pairing they had learned. Participants first completed 20 practice trials, after which the experimental trials began. The experimental phase consisted of 200 trials. Of these, 100 trials were matching trials and 100 were non-matching trials. Half of the matching trials involved the self-associated shape–label pair, and half involved the stranger-associated pair. Trials were presented in a randomized order for each participant. Each trial began with a fixation cross displayed at the centre of the screen for 500 ms, followed by the presentation of a shape (either a square or a triangle) and a label (either “YOU” or “STRANGER”) for 100 ms. The shape measured 0.3 × 0.3 height units, while the label was displayed in Arial font with a letter height of 0.06 height units. Both stimuli were located 0.3 height units above and below the vertical centre, respectively, and this spatial arrangement was kept constant throughout the task. After stimulus offset, a blank screen was presented for 1500 ms, during which participants could respond. The blank screen lasted the full 1500 ms regardless of when the response occurred, after which feedback indicating response accuracy was displayed for 500 ms (see Fig. 3). The same procedure was administered at both S2 and S3. At S3, participants completed the learning phase again with the same shape–label assignment they had been given at S2.

### Data analysis

Statistical analyses were conducted using R (version 4.5.1; R Core Team, 2025). For each session, participants whose identification codes could not be uniquely resolved were removed prior to all subsequent analyses. Specifically, this resulted in the exclusion of 3 participants at the first session (of 149 collected; usable *N* = 146), 3 pairs of duplicate codes were identified at the second session (6 excluded; usable *N* = 113), and 4 pairs at the third session (8 excluded; usable *N* = 89). Additional task-specific exclusion criteria are described in the corresponding scoring sections below.

### SE-IAT scoring

The SE-IAT D-score measures the strength of the implicit association between self and positive attributes relative to self and negative attributes. It is computed as the standardized difference in mean RT between incongruent blocks (self + negative, other + positive) and congruent blocks (self + positive, other + negative), such that positive values indicate a stronger implicit association between self and positive valence.

SE-IAT D-scores were computed using the improved scoring algorithm described by Greenwald et al. (2003). This algorithm incorporates data from both practice and test phases, replaces error latencies with the block mean plus a 600-ms penalty, and scales the resulting difference by each participant’s pooled standard deviation. Trials with latencies exceeding 10,000 ms were removed (fewer than 0.1% of all recorded trials at each time point; no trials excluded at the second session). Participants with more than 10% of trials faster than 300 ms were excluded; for the second session, one participant met this criterion (34% of trials below 300 ms) and was removed. Trials with latencies below 200 ms were additionally removed to eliminate anticipatory responses (fewer than 0.1% of all recorded trials at each time point). Final samples for SE-IAT analyses were *N* = 146 at the first session and *N* = 112 at the second session.

### NLT scoring

NLT scores were computed as the difference between the mean rating assigned to participants’ own name and surname initials and the mean rating assigned to all other letters (Nuttin, 1985). Higher scores indicate greater preference for own initials, reflecting higher implicit self-esteem. No additional exclusions were applied. The final sample was *N* = 146.

### RSES scoring

RSES total scores were computed by summing responses across the 10 items after reverse-coding negatively worded items (Rosenberg, 1989; Prezza et al., 1997). Scores range from 0 to 30, with higher values indicating higher explicit self-esteem. No additional exclusions were applied. Final samples were *N* = 146 for the first session and *N* = 113 for the second session.

### SPE scoring

Following the multiverse reliability assessment by Liu et al. (2025), which identified RT-based indices with a stranger baseline as the most reliable measure of the SPE at the individual level, we quantified the SPE using RTs. Specifically, the SPE was computed for each participant as the difference between mean RT for stranger-matching trials and mean RT for self-matching trials (RT stranger – RT self). Positive values indicate faster responses to self-related stimuli.

### Statistical approach

Relationships among implicit SE (SE-IAT, NLT), explicit SE (RSES), and the SPE were examined using Pearson correlation coefficients. Cross-sectional correlations were computed separately for each time point; longitudinal correlations were calculated on participants who completed multiple sessions. Test-retest reliability was assessed for measures administered at multiple time points. To control for multiple testing, a False Discovery Rate correction (FDR; Benjamini & Hochberg, 1995) was applied across all the correlation analyses involving SE measures and the SPE.

## Results

### SPE

RT analyses focused exclusively on correctly responded matching trials; missed (5.13% of matching trials at the second session; 4.64% of matching trials at the third session) and incorrect responses (10.31% of matching trials at the second session; 8.41% of matching trials at the third session) were excluded. Outliers exceeding ± 3 SD from individual condition means were removed (0.86% trials at the second session; 1.27% trials at the third session) on a participant-wise basis. Participants with fewer than 50 valid matching trials (out of a maximum of 100) were excluded (*n* = 7 at the second session; *n* = 5 at the third session). This threshold served as a data-quality criterion: participants losing more than half of their informative trials to combined errors, misses, or outlier responses were considered unlikely to have engaged meaningfully with the task. A sensitivity analysis confirmed that the pattern of correlational findings was robust to this choice across thresholds ranging from 0 to 70 valid trials. Final samples for SPE analyses were *N* = 106 at the second session and *N* = 84 at the third session.

Mean RTs for correctly responded matching trials in the second session were lower in the “YOU” condition (*M* = 687 ms, *SD* = 104 ms) compared to the “STRANGER” condition (*M* = 856 ms, *SD* = 131 ms), indicating a robust SPE. Mean accuracy was higher in matching trials related to the self (M = 0.94; SD = 0.10), compared to matching trials in the non-self condition (M = 0.83; SD = 0.13). In the third session, the same pattern was observed. Mean RTs were lower in the “YOU” condition (M = 687 ms, SD = 146 ms) than in the “STRANGER” condition (M = 812 ms, SD = 163 ms). Accordingly, mean accuracy for matching trials was higher in the self condition (M = 0.94; SD = 0.10), rather than in the non-self condition (M = 0.87; SD = 0.14). To formally test the SPE at each session, RTs from correctly responded matching trials were modelled using a Generalized Linear Mixed Model (GLMM) with identity (self vs. stranger) as fixed effect, random intercept for participants, and a Gamma family with log link function. The effect of identity was statistically significant for both the second (*β* = 0.22, *SE* = 0.005, *z* = 45.13, *p* <.001) and the third session (*β* = 0.17, *SE* = 0.005, *z* = 31.11, *p* <.001), confirming a robust SPE in both sessions.

### Self-esteem measures

After applying the exclusion criteria described above, 146 participants were retained for the first-session analyses. Descriptive statistics for all SE measures are summarised in Table 2. SE-IAT *D*-scores revealed a positive mean (*M* = 0.58, *SD* = 0.32), indicating that participants, on average, held stronger implicit associations between self and positive (vs. negative) attributes. NLT scores were also predominantly positive (*M* = 2.04, *SD* = 1.60), with 135 of 146 participants exhibiting a preference for their own initials. RSES total scores (*M* = 16.97, *SD* = 5.21) fell within the typical range for non-clinical samples (Prezza et al., 1997), though considerable inter-individual variability was observed.

**Table 2:** Descriptive statistics for self-esteem measures

Measure	Session	*N*	M	SD
SE-IAT D-score	1	146	0.58	0.32
SE-IAT D-score	2	112	0.50	0.30
NLT score	1	146	2.04	1.60
RSES score	1	146	16.97	5.21
RSES score	2	113	17.68	4.64

The table reports the descriptive statistics for self-esteem measured at the first and second session. For all measures, higher scores indicate higher self-esteem. *SE-IAT* Self-Esteem Implicit Association Test, *NLT* Name Letter Test, *RSES* Rosenberg Self-Esteem Scale

SE-IAT *D*-scores collected in the second session (*M* = 0.50, *SD* = 0.30) remained positive, again reflecting a general tendency towards positive implicit self-evaluation. RSES total scores collected in the second session (*M* = 17.68, *SD* = 4.64) were likewise consistent with the values of the first session (see Table 2).

### Correlational analyses

#### Cross-sectional correlations at the first session

All bivariate correlations among study variables are reported in Table 3. The SE-IAT *D*-score was not significantly associated with either the NLT score (*r* =.00, *p* =.977) or the RSES total (*r* = −.01, *p* =.947). Similarly, the NLT showed a non-significant correlation with the RSES (*r* = −.03, *p* =.726). A paired-samples *t*-test was conducted to examine whether mean explicit SE changed across the two sessions in which the RSES was administered (*N* = 90). Mean RSES scores did not differ significantly between S1 and S2, *t*(89) = − 1.29, *p* =.200, *M*diff = − 0.37, 95% CI [− 0.93, 0.20]. Similarly, a paired-samples *t*-test was used to test whether mean implicit SE changed across the two sessions in which the SE-IAT was administered. SE-IAT *D*-scores showed a small but significant decrease from S1 to S2, *t*(89) = 2.47, *p* =.016, *M*diff = 0.09, 95% CI [0.02, 0.17].

**Table 3:** Bivariate correlations among study variables

Measure	1. SE-IAT S1	2. NLT S1	3. RSES S1	4. SE-IAT S2	5. RSES S2	6. SPE S2	7. SPE S3
1. SE-IAT S1	—						
2. NLT S1	0.00	—					
3. RSES S1	−0.01	−0.03	—				
4. SE-IAT S2	0.30	—	—	—			
5. RSES S2	—	—	0.86	0.10	—		
6. SPE S2	0.09	0.04	0.12	0.08	0.28	—	
7. SPE S3	0.00	0.09	0.35	0.06	0.32	0.49	—

Values are Pearson correlation coefficients computed with pairwise deletion. Sample sizes vary across cells due to the longitudinal design and specific exclusion criteria detailed in the main text (range: *N* = 65–146). *SE-IAT* Self-Esteem Implicit Association Test (D-score), *NLT* Name Letter Test, *RSES* Rosenberg Self-Esteem Scale, *SPE* Self-Prioritization Effect. S1, S2, and S3 refer to the first, second, and third testing sessions, respectively

#### Correlations between the SPE and SE measures

**Fig. 4:**
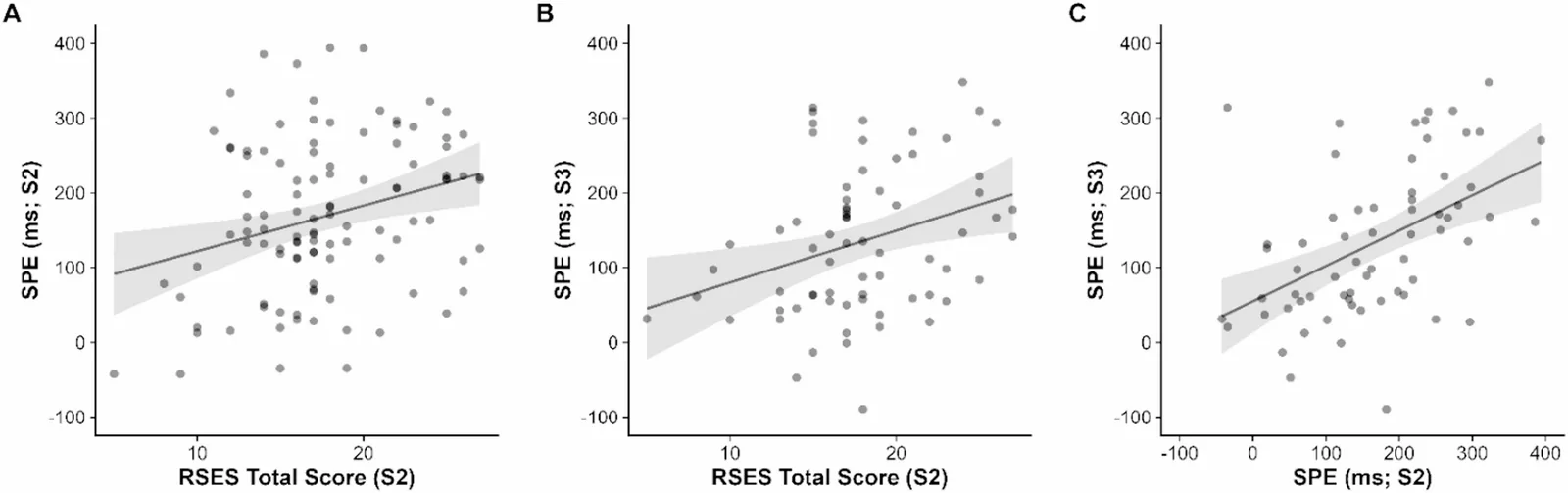
RSES–SPE associations and temporal stability of the SPE. Scatter plots showing the association between RSES in the second session and the SPE (Panels **A **and **B**) and the temporal stability of the SPE (Panel **C**). Panel A shows the concurrent association in the second session (*r* =.28, *N* = 106). Panel B shows the cross-time association between RSES scores measured in the second session and SPE measured in the third session (*r* =.32, *N* = 69). RSES = Rosenberg Self-Esteem Scale; SPE = Self-Prioritization Effect. Solid lines represent linear regression fits; shaded areas denote 95% confidence intervals. Panel C shows the temporal stability of the SPE measured in the second and third session (*r* =.49, *N* = 65). The solid line represents the linear regression fit; the shaded area denotes the 95% confidence interval

The adjusted *α* level for the number of comparisons according to the FDR procedure for multiple testing (Benjamini & Hochberg, 1995) was *p_FDR* = .023. Cross-time correlations between the SPE obtained in the second session and SE measures collected in the first session were computed for the subset of participants who completed both sessions (*N* = 84). The SPE in the second session did not correlate significantly with any of the first session SE measures: SE-IAT *D*-score (*r* =.09), NLT (*r* =.04), or RSES (*r* =.12). A significant positive correlation emerged between RSES scores collected in the first session and the SPE in the third session (*r* =.35, *N* = 66), indicating that explicit SE predicted the self-prioritization advantage approximately two weeks later, even when measured in a context independent of the matching task. When examining the concurrent second-session measures, the SPE was not significantly correlated with the SE-IAT *D*-score (*r* =.08, *N* = 105). However, a significant positive association emerged between the SPE and the RSES (*r* =.28, *N* = 106), indicating that participants reporting higher explicit SE in a given experimental session displayed a larger self-prioritization advantage in the same session (see Fig. 4, Panel A). A similar, statistically significant, positive association was also found for explicit SE and the self-prioritization advantage in the subsequent session (i.e., RSES at S2 predicting the SPE at S3: *r* =.32, *N* = 69; see Fig. 4, Panel B).

#### Temporal stability and longitudinal associations

Test–retest correlations were computed for measures administered twice. The RSES exhibited high temporal stability (*r* =.86, *p* <.001), whereas the SE-IAT *D*-score showed a modest but significant correlation across the two testing sessions (*r* =.30, *p* =.004). The SPE likewise showed moderate stability between the second and third session (*r* =.49, *p* <.001, *N* = 65; see Fig. 4 Panel C). Although cross-session changes were not the main focus of the present study, for the sake of completeness, a GLMM (Gamma family, log link) with identity (self vs. stranger) and session (S2 vs. S3) as fixed effects and a random intercept for participant was used to test whether the magnitude of the SPE changed across sessions in the 65 participants who completed the matching task at both sessions. The identity × session interaction was significant (β = −0.03, *z* = − 3.90, *p* <.001): the SPE was smaller at S3 than at S2 (stranger responses were 24.3% slower than self responses at S2 and 20.2% slower at S3), although the change was modest and the stability across sessions was moderate. Additionally, to formally examine whether the strength of the RSES–SPE association varied across assessments, we compared the relevant dependent correlations using Williams’ tests, with FDR correction (Benjamini & Hochberg, 1995) applied across the three comparisons; confidence intervals for the difference between correlations were computed following the method reported by Zou (2007). Since these comparisons require complete data on all variables involved, they were computed on the corresponding complete-case subsamples (*N* = 84, 58, and 65, respectively), so that the coefficients reported here differ slightly from the corresponding values in Table 3. The association between the SPE at S2 and the RSES measured at S1 was significantly weaker than the association between the SPE at S2 and the RSES measured concurrently at S2 (*r* =.12 vs. *r* =.26), *t*(81) = − 2.67, *p*_FDR = .028, 95% CI for the difference [−0.26, − 0.04]. By contrast, the association between RSES at S1 and the SPE did not differ significantly between S2 and S3 (*r* =.17 vs. *r* =.38), *t*(55) = − 1.61, *p*_FDR = .170, 95% CI [− 0.46, 0.05], and the association between RSES at S2 and the SPE was virtually identical at S2 and S3 (*r* =.31 vs. *r* =.32), *t*(62) = − 0.05, *p*_FDR = .959, 95% CI [− 0.24, 0.23]. Thus, although the strength of the RSES–SPE association was not entirely uniform across assessments, the prospective associations remained positive and no evidence emerged for a reversal of the relationship.

## Discussion

The current study explored the relationship between the SPE and both implicit and explicit SE using a longitudinal design with three measurement sessions. During the first session, participants completed assessments of implicit SE (SE-IAT, NLT) and explicit SE (RSES). During the second session, participants performed the matching task assessing the SPE, followed by the SE-IAT and RSES. During the third session, participants were only administered the matching task.

The results confirmed a robust SPE at both measurement sessions, with participants showing substantially faster RTs and higher accuracy for self-matching trials compared to stranger-matching trials. Notably, the SPE demonstrated moderate temporal stability across sessions, aligning with the findings reported by Liu et al. (2025) for RT-based SPE measures using the baseline provided by trials related to the stranger. As regards the correlation between the SPE and SE measures, our results revealed different patterns of findings for implicit and explicit forms of self-evaluation. The two implicit SE measures, the SE-IAT and NLT, showed no significant correlations with the SPE, irrespective of whether assessed concurrently (in the second session) or cross-temporally. In contrast, explicit SE as measured by the RSES administered during the second session exhibited a significant positive association with the SPE both concurrently (*r* =.28, *p* =.003) and prospectively with the SPE measured in the subsequent testing session (*r* =.32, *p* =.007). These findings indicate that individuals reporting higher explicit self-worth displayed larger self-prioritization advantages, and that this relationship was not confined to a single measurement occasion.

RSES scores measured in the first administration were not significantly correlated with the SPE at the second session (*r* =.12, *p* =.286), although the pattern was in the direction of a positive association. Crucially, RSES scores measured at S1 did significantly predict the SPE measured at the third session (*r* =.35, *p* =.005). This finding, coupled with the significant correlations between RSES scores measured at S2 and SPE at S2, and RSES scores measured at S2 and SPE at S3, suggests that explicit SE is positively associated with the SPE when assessed concurrently or prospectively.

Overall, our pattern of results diverges from the null finding reported by Schäfer and Frings (2019), who examined the association between explicit SE, measured using the RSES and the SPE measured using a matching task. Several methodological differences may play a role in this discrepancy, which include, for instance, the number of experimental trials and the baseline for assessing the SPE. In this regard, recent evidence suggests that the choice of the baseline may moderate both the magnitude and reliability of the SPE, with “stranger” producing more consistent estimates than alternative baselines (Liu et al., 2025). One possibility is that a more reliable SPE measure would increase the likelihood to observe correlations with other constructs. On the same line, the results of the present study differ from the findings reported by Mou and Ding (2026), who explored the relationship between explicit SE and the SPE using a voice-label matching task in which unfamiliar voices were arbitrarily associated with the self, a friend, or a stranger. Consistent with Schäfer and Frings (2019), explicit SE was not significantly associated with self-bias derived from neutral vocalizations. The discrepancy between our findings and those reported by Mou and Ding (2026) may be due to methodological factors and also reflect genuine differences across stimulus modalities in how self-associated information is processed and in the degree to which explicit self-evaluation modulates such processing.

Our findings are consistent with those reported by Tan and Li (2025), who used a comparable shape–label matching paradigm in a sample of children aged 9 to 11 and found that explicit SE positively predicted the magnitude of the SPE, whereas implicit SE did not emerge as a significant predictor.

The observed positive correlation between explicit SE and the SPE raises important questions for theoretical accounts of the effect. Specifically, the selective association with explicit SE suggests that higher-order self-evaluative processes can, at least partially, be associated with the strength of the SPE. This finding is broadly consistent with the positive-association theory of self-advantage, which attributes the processing advantage of self-related stimuli to the positive valence implicitly linked to the self (Ma & Han, 2010; but see Lee et al., 2024). A related account is offered by Sui et al. (2012), who proposed that neutral stimuli associated with the self gain social salience that enhances their processing at early perceptual stages. The present findings are compatible with the possibility that such salience is not fixed or predetermined but rather varies as a function of how positively individuals consciously evaluate themselves, suggesting that self-related stimuli are perceived as more perceptually salient by individuals who hold a more positive explicit self-view. This possibility should nonetheless be considered with caution. The matching task requires participants to both keep the shape–label associations active in working memory and perform an explicit match/mismatch judgment. The association between explicit SE and the SPE could therefore emerge at more central stages (e.g., attentional, motivational, decisional), rather than reflecting a modulation of perceptual processing per se. This interpretation is consistent with evidence that self-prioritization is not a fixed, purely perceptual phenomenon, but operates across multiple stages of processing and is sensitive to how self-relevant stimuli are evaluated (Constable et al., 2021; Hu et al., 2020).

The question of whether the association operates at a perceptual or at a more central level need not be resolved in either direction. A useful framework for reconciling these possibilities comes from the distinction between the minimal and the narrative self proposed by Gallagher (2000). According to this perspective, the minimal self refers to a pre-reflective, low-level distinction between self and non-self, whereas the narrative self refers to the integration of autobiographical information and self-evaluative contents. The matching task has been previously regarded as a window onto the minimal self, given its reliance on rapid behavioural responses to newly learned, neutral shape–label associations (Schäfer & Frings, 2019; Sui et al., 2012). Accordingly, Schäfer and Frings (2019) interpreted the absence of a correlation between explicit SE and the SPE in their study as evidence of a functional independence between the two constructs, framing explicit SE at the level of the narrative self and the SPE at the level of the minimal self. The present findings lead to a partially different conclusion. The positive association between explicit SE and the SPE observed both concurrently (at the second session) and prospectively (e.g., explicit SE measured at the second session and SPE measured at the third session) suggests that the rapid, pre-reflective prioritization captured by the matching task is not fully independent of higher-order self-evaluations, providing empirical evidence relevant to the open issue recently raised by Hommel (2018) concerning the possible relationship between minimal and narrative aspects of self-representation. In other words, low-level and higher-order aspects of self-representation concerning the SPE may be more closely interconnected than previously assumed.

Interestingly, implicit SE measures showed no significant associations with the SPE. This pattern may reflect genuine construct independence: implicit self-evaluations, as captured by the SE-IAT and NLT, may tap different aspects of self-representation than those engaged during the matching task. However, measurement limitations may also have played a role. The modest test-retest reliability of the SE-IAT observed in our data (*r* =.30 between the first and the second session) is consistent with broader concerns about the psychometric properties of implicit measures and may have attenuated our ability to detect true associations. Furthermore, the SE-IAT and NLT were neither significantly correlated with each other nor with the RSES, consistent with prior evidence that different implicit measures often show weak convergence (Bosson et al., 2000). This lack of convergent validity complicates the interpretation of these results, leaving open whether they reflect true independence between implicit SE and the SPE or insufficient measurement precision.

Crucially, the present results should be considered in light of some methodological considerations. First, the number of participants decreased across sessions, from 146 at the first session to 106 at the second session and 84 at the third session, reducing statistical power for longitudinal analyses and potentially introducing a selection bias. However, the replication of the RSES–SPE correlation across both the second and third session provides some converging evidence for the robustness of the finding. Moreover, although the reduction in sample size represents a limitation, the current study is among the few to provide longitudinal data on the SPE, addressing the need for repeated assessments highlighted by recent reliability analyses (Liu et al., 2025). A further consideration concerns the fixed order of administration. Since the order of task execution was not counterbalanced, a choice that was methodologically motivated (see Methods), differences between the baseline and the concurrent SE measures may partly reflect task-order or practice-related influences, in addition to genuine engagement with the matching task. This consideration is particularly relevant for the interpretation of the association between the RSES and the SPE measured within the second session, which was stronger than the association between the RSES collected at the first session and the SPE measured at the second session, as confirmed by the formal comparison reported in the Results section. Since at the second session the RSES was administered after the matching task, the stronger association observed within that session may partly reflect the shared session context, rather than the SE–SPE relationship alone. Notably, however, explicit SE collected at the first session, in a context independent of the influence of the matching task, still predicted the SPE measured at the third session two weeks later. This suggests that the association between SE and SPE is not confined to a single measurement occasion. Additionally, it is worth noting that the correlational nature of the design precludes causal inferences. Although our data show that higher explicit self-esteem is reliably associated with a larger SPE, they cannot establish whether self-esteem plays a causal role in shaping self-prioritization. Addressing this question would require experimental rather than observational approaches. Manipulating self-evaluative states, for instance through self-affirmation interventions or providing bogus feedback (e.g., Heatherton & Polivy, 1991), and examining whether such manipulations produce corresponding changes in the magnitude of the SPE would provide a more direct test of this possibility and represents a valuable direction for future research.

In conclusion, the current study provides evidence that explicit SE is positively associated with the SPE, diverging from prior work that reported no such relationship in adult samples (Mou & Ding, 2026; Schäfer & Frings, 2019). This association emerged not only concurrently within the second session but also prospectively across sessions, suggesting that it is not confined to a single measurement occasion, although the stronger concurrent association may have been partly influenced by the shared session context. Notably, no significant associations were found between the SPE and implicit SE measures, pointing to a dissociation between propositional and automatic forms of self-evaluation in their relationship with self-prioritization. Future research should employ different experimental designs to examine whether changes in explicit SE produce corresponding changes in the SPE.

## Data Availability

No datasets were generated or analysed during the current study.
